# Topiramate induced acute transient myopia: a case report

**DOI:** 10.4076/1757-1626-2-7430

**Published:** 2009-07-03

**Authors:** Sandra D Gawley

**Affiliations:** St Paul’s Eye Unit, The Royal Liverpool University HospitalPrescot Street, Liverpool, MerseysideUK

## Abstract

**Introduction:**

Topiramate is a sulfamate-substituted monosaccharide mainly used to treat epilepsy in children and adults and for prophylaxis of migraine. This article describes a case of topiramate induced acute transient myopia. The underlying mechanism and management is discussed.

**Case presentation:**

A 34-year-old female complained of sudden onset of blurred vision, 9 days prior to this she had commenced topiramate therapy for migraine prophylaxis. Visual acuity was reduced to 6/36 right eye and 2/60 left eye. Examination revealed ocular anatomical and myopic refractive changes which resolved quickly following discontinuation of the drug.

**Conclusion:**

Ophthalmologists need to be aware of the potential ocular side effects of topiramate. Although relatively rare prompt recognition is key so appropriate management can be instituted.

## Case presentation

A 34-year-old female complained of sudden onset of blurred vision. She attended her optician who noted a large myopic shift from –1.00/+0.50 × 90 right eye, −0.75/+0.25 × 95 left eye to –3.50/+0.50 × 90 right eye, −3.50/+0.25 × 95 left eye. Oral topiramate treatment had been commenced for migraine prophylaxis 9 days prior to the onset of her symptoms at a dose of 25 mg once daily (OD), increased 7 days later to 50 mg OD. On examination Snellen visual acuity with her glasses on was 6/36 right eye, 2/60 left eye, with full new myopic correction vision was 6/6 bilaterally. Both eyes were white and quiet but anterior chambers were shallow and the iris and lens were bowed forward. Intraocular pressures (IOP) were 18 mHg and Gonioscopy revealed 360° Shaffer Grade 1. No choroidal effusions were seen by indirect ophthalmoscopy but B-scan showed a small separation between the choroidal and scleral layers bilaterally in keeping with small effusions. Given the patient had discontinued the topiramate therapy herself and as IOPs were normal no treatment was instituted. At review 2 days later vision with her glasses on had improved to 6/9 right eye, 6/36 left eye. The anterior chambers were deep, IOP normal and the iris and lens had returned to a normal position and configuration. Two weeks later repeat Gonioscopy showed 360^o^ Shaffer Grade 4 angles, B-scan ultrasound showed resolution of the choroidal effusions and refraction was –0.75/+0.50 × 100 right eye, −0.75/+0.50 × 90 left eye, with this correction vision was 6/5 bilaterally.

## Discussion

As well as epilepsy and migraine topiramate has also been used to treat depression, neuropathic pain, as a weight reduction agent and for bipolar disorder. Case reports on ocular side effects of this drug date back to 2001 [1-3]. In September 2001 Ortho-McNeil Pharmaceuticals sent out a safety alert to healthcare professionals indicating 23 cases of secondary angle-closure glaucoma related to topiramate use based on post-marketing experience in more than 825,000 patients. (Hulihan J: Important drug warning [letter]. Available at: http://www.fda.gov/medwatch/SAFETY/2001/topamax_deardoc.PDF).

The majority of reported adverse events have occurred in female patients (up to 89%) [4]. Ocular side effects have also been reported in children [5]. In the “certain” category of the World Health Organisation classification system adverse ocular side effects associated with topiramate include abnormal vision, acute IOP elevation, acute myopia (up to 8.75 dioptres), diplopia, nystagmus and shallow anterior chamber with angle-closure. “Probable/likely” include blepharospasm, myokymia, oculogyric crisis, suprachoroidal effusions and “possible” are congenital ocular abnormalities, periorbital oedema and scleritis [6]. High frequency ultrasound biomicroscopy, anterior segment ocular coherence tomography and B-scan ultrasound have helped establish and document the underlying mechanism of the myopia and angle-closure glaucoma [7-9] – uveal effusions and ciliary body oedema result in antero-lateral rotation of the ciliary body, anterior displacement of the lens-iris diaphragm which contributes to the myopic shift, anterior chamber shallowing and secondary appositional angle closure. The effusion and oedema also lead to relaxation of the lens zonules resulting in thickening of the lens further narrowing the angle. Though the exact mechanism is unclear the fluid movement leading to effusions is thought to be related to drug induced changes in membrane potential [8]. In reported cases of angle-closure glaucoma topiramate doses varied from 50 mg or less to 100 mg or more, 5 reported cases were precipitated within hours after doubling the dose, 85% of cases occurred in the first 2 weeks of treatment with the drug [10].

Fraunfelder et al [10] advise the following management strategy for topiramate-associated angle-closure glaucoma:

Stoppage of the drug in the first instance, the prescribing doctor should be consulted.

Medical therapy such as oral medications and aqueous suppressants should be given.

Laser iridotomy or peripheral iridectomy are not helpful as topiramate angle closure is not pupil block related.

Topical miotics may be contraindicated as they could precipitate a relative pupil block.

Topical cycloplegic agents may be given as they possibly lower IOP by retracting ciliary processes.

Care should be taken with acetazolamide as it is also a sulfa-based drug and has been reported to cause angle-closure glaucoma in a similar manner to topiramate [11].

In this case the topiramate induced anatomical changes stopped short of inducing angle-closure glaucoma. The rapid onset of visual loss secondary to the myopia is understandably distressing to the patient and it is helpful to be able to provide some guidance on prognosis – as the mean plasma elimination half life of the drug is about 21 hours [12], rapid visual recovery usually occurs although in some cases it can take several weeks [10]. If unrecognised as a drug-related event serious outcomes could occur (7 cases of permanent visual loss following angle-closure glaucoma have been reported) [10]. Ocular examination before starting topiramate cannot identify eyes at risk [8]. Patients commencing topiramate should therefore be advised to immediately report any symptoms of eye pain or blurred vision especially in the first few weeks of treatment.

## Conclusion

Ophthalmologists need to be aware of the potential ocular side effects of topiramate. Although relatively rare prompt recognition is key so appropriate management can be instituted and visual outcomes maximised.

## Abbreviation

IOP: Intraocular pressures
